# Prognostic factors related to dendritic cell vaccines on patients with advanced non-small cell lung cancers: a multicenter analysis

**DOI:** 10.1186/2051-1426-3-S2-P451

**Published:** 2015-11-04

**Authors:** Hidenori Takahashi, Shigetaka Shimodaira, Masahiro Ogasawara, Masanori Kobayashi, Hirofumi Abe, Kazuhiro Nagai, Sunichi Tsujitani, Masato Okamoto, Yuji Morita, Yoshikazu Yonemitsu

**Affiliations:** 1 Seren Clinic Fukuoka, Fukuoka, Japan; 2 Cell Processing Center, Shinshu University Hospital, Matsumoto, Nagano, Japan; 3 Department of Hematology, Sapporo Hokuyu Hospital, Sapporo, Hokkaido, Japan; 4 Seren Clinic Nagoya, Nagoya, Aichi, Japan; 5 Seren Clinic Kobe, Kobe, Hyogo, Japan; 6 Transfusion and Cell Therapy Unit, Nagasaki University Hospital, Nagasaki, Japan; 7 Cancer Center, Tottori University Hospital, Yonago, Tottori, Japan; 8 Department of Advanced Immunotherapeutics, Kitasato University School of Pharmacy, Tokyo, Japan; 9 Seren Clinic Tokyo, Tokyo, Japan; 10 R&D Laboratory for Innovative Biotherapeutics, Graduate School of Pharmaceutical Sciences, Kyushu University, Fukuoka, Japan

## Objective

Dendritic cell (DC)-based cancer vaccines may have a significant benefit to patients with advanced non-small cell lung cancers (NSCLCs). However, variations among clinical studies make it difficult to compare clinical outcomes. J-SICT DC vaccine study group is composed of the multi-medical centers in Japan, has provided DC vaccines as a compassionate use under the unified regimens for cell production and patient treatment, and published a number of clinical data of patients treated with DC vaccines. A single medical institution of this group has recently published a potentially beneficial effect of DC vaccines on overall survival of 62 patients with advanced NSCLCs[1]. Here we extended the findings to 260 patients with advanced NSCLCs who treated among 6 centers of this group.

## Methods

Of 337 patients who met the inclusion criteria, 260 patients who received by weekly more than 5-times of peptide-pulsed DC vaccines were analyzed.

## Results

No serious adverse event related to DC vaccination. The mean survival time from diagnosis was 33.0 months (95%CI=27.9–39.2, 85.5% in 1-year and 66.4% in 2-years), and that from the first vaccination was 13.8 months (95%CI=11.4–16.8, 53.5% in 1-year and 36.1% in 2-years). Similar to our previous findings obtained in the analysis for advanced pancreatic cancer[2], 30 mm and more in diameter of erythema reaction at the injected site was identified as the strongest factor correlating to the overall survival from 1^st^ vaccine (> 30 mm: MST=20.4 months, n=135 and < 30 mm: MST=8.8 months, n=122, p < 0.0001) in uni- and multivariate analyses. Importantly, the size of erythema reaction was significantly correlated with ECOG-PS, neutrophil/lymphocyte (N/L) ratio, hemoglobin, C-reactive protein, and fever (over 38 ^o^C) at the time of leukapheresis, suggesting that the good physical condition as well as less inflammatory reaction might be important to induce stronger reaction against DC vaccines.

## Conclusions

This is the first report of a multicenter clinical study suggesting the feasibility and possible clinical benefit of DC vaccines in patients with advanced NSCLCs. These findings suggest the potential responders against DC vaccines, and need to be addressed in well-controlled prospective randomized trials.
